# IFNγ at the early stage induced after cryo-thermal therapy maintains CD4^+^ Th1-prone differentiation, leading to long-term antitumor immunity

**DOI:** 10.3389/fimmu.2024.1345046

**Published:** 2024-05-17

**Authors:** Junjun Wang, Yue Lou, Shicheng Wang, Zelu Zhang, Jiaqi You, Yongxin Zhu, Yichen Yao, Yuankai Hao, Ping Liu, Lisa X. Xu

**Affiliations:** School of Biomedical Engineering and Med-X Research Institute, Shanghai Jiao Tong University, Shanghai, China

**Keywords:** cryo-thermal therapy, interferon-γ, CD4^+^ Th1 cells, myeloid-derived suppressor cells, interleukin-1β

## Abstract

**Introduction:**

Recently, more and more research illustrated the importance of inducing CD4^+^ T helper type (Th)-1 dominant immunity for the success of tumor immunotherapy. Our prior studies revealed the crucial role of CD4^+^ Th1 cells in orchestrating systemic and durable antitumor immunity, which contributes to the satisfactory outcomes of the novel cryo-thermal therapy in the B16F10 tumor model. However, the mechanism for maintaining the cryo-thermal therapy-mediated durable CD4^+^ Th1-dominant response remains uncovered. Additionally, cryo-thermal-induced early-stage CD4^+^ Th1-dominant T cell response showed a correlation with the favorable prognosis in patients with colorectal cancer liver metastasis (CRCLM). We hypothesized that CD4^+^ Th1-dominant differentiation induced during the early stage post cryo-thermal therapy would affect the balance of CD4^+^ subsets at the late phase.

**Methods:**

To understand the role of interferon (IFN)-γ, the major effector of Th1 subsets, in maintaining long-term CD4^+^ Th1-prone polarization, B16F10 melanoma model was established in this study and a monoclonal antibody was used at the early stage post cryo-thermal therapy for interferon (IFN)-γ signaling blockade, and the influence on the phenotypic and functional change of immune cells was evaluated.

**Results:**

IFNγ at the early stage after cryo-thermal therapy maintained long-lasting CD4^+^ Th1-prone immunity by directly controlling Th17, Tfh, and Tregs polarization, leading to the hyperactivation of Myeloid-derived suppressor cells (MDSCs) represented by abundant interleukin (IL)-1β generation, and thereby further amplifying Th1 response.

**Discussion:**

Our finding emphasized the key role of early-phase IFNγ abundance post cryo-thermal therapy, which could be a biomarker for better prognosis after cryo-thermal therapy.

## Introduction

Immunotherapy has developed as a pivotal component of tumor therapies. CD8^+^ T cell-mediated tumor killing is regarded as a key for tumor regression, but recent research presented the essential role of Interferon (IFN)-γ-producing CD4^+^ T helper(Th)-1 cells in mediating long-term antitumor immune protection (1, 2). CD4^+^ Th1 cells orchestrate tumor killing by stimulating the production of major histocompatibility complex (MHC) class II on tumor cells, facilitating the accumulation of innate and adaptive immune cells with enhanced cytotoxicity function (3). Inducing CD4^+^ Th1-dominant immunity is the main target for tumor immunotherapy (4).

Previously, an innovative cryo-thermal therapy was established for primary tumor ablation, involving rapid liquid nitrogen (LN_2_) freezing followed by radiofrequency heating, which maximizes the release of tumor antigens and damage-associated molecular patterns (DAMPs) to establish CD4^+^ Th1-orchestrated tumor-specific immunity, and enhances the cytotoxicity of macrophages, CD8^+^ T cells, and natural killer (NK) cells, thereby eliminating micrometastases of tumors and improving long-term survival in the animal tumor models (5–8). However, the mechanism of how to maintain the cryo-thermal therapy-activated long-term CD4^+^ Th1-dominant response was uncovered.

CD4^+^ T cells undergo differentiation into distinct subsets characterized by the secretion of diverse cytokines and the acquisition of specific functions in response to a complex interplay between cytokine networks, antigen presentation, and costimulatory signaling (9). CD4^+^ Th1 cells, the major antitumor immune response regulator, promote an IFNγ-abundant immunostimulatory environment to maintain their dominant differentiation. The autocrine IFNγ signaling induces T-bet expression, a typical transcriptional factor of CD4^+^ Th1 cells (1). Meanwhile, IFNγ prevents the polarization of Th17, T follicular helper (Tfh) cells, and regulatory T cells (Tregs) by downregulating their functional transcriptional factors (10–12). Moreover, IFNγ triggers the coexpression of T-bet in Th17 subsets, Tfh cells, and Tregs, thus inducing their repolarization into CD4^+^ Th1 cells and driving fragility (13–15). Previous research of cryo-thermal therapy application in clinical colorectal cancer liver metastasis (CRCLM) patients promotes early-stage differentiation of CD4^+^ Th1 cells and significantly extended the progression-free survival (PFS) of patients (16), which leads to the suggestion that the maintaining of persistent CD4^+^ Th1-dominant immunity may attribute to early-stage IFNγ abundance mediated by cryo-thermal therapy-induced CD4^+^ Th1-dominant differentiation.

To test, in this study, a subcutaneous B16F10 tumor model was established, and IFNγ, the effector cytokine of CD4^+^ Th1 cells, was *in vivo* neutralized post cryo-thermal therapy with a monoclonal antibody (mAb). Results demonstrated the importance of Th1-prone CD4^+^ T cells elicited early post cryo-thermal therapy, accompanied by an increased level of serum IFNγ in maintaining CD4^+^ Th1-dominant differentiation through preventing Th17, Tfh, and Tregs differentiation, as well as inducing MHCII, CD86, and Interleukin (IL)-1β expressive hyperactive Myeloid-derived suppressor cells (MDSCs), which further maintained Th1-priority polarization subsequently. These studies reflect the key role of early-stage IFNγ post cryo-thermal therapy in establishing long-term CD4^+^ Th1-dominant immunity, which potentially serves as a promising biomarker of prognosis.

## Methods

### Cell cultivation and mouse melanoma model establishment

B16F10 mouse melanoma cell line was provided by Professor Weihai Yin at Med-X Research Institute, Shanghai Jiao Tong University. The female C57BL/6 mice were sourced from Shanghai Slaccas Experimental Animal Co., Ltd. (Shanghai, China). The cell culture and mouse B16F10 melanoma model establishing methods were following previous research protocol (6). To establish the subcutaneously (s.c.) melanoma tumor model, mice aged 6–8 weeks were injected with nearly 5×10^5^ B16F10 melanoma cells into the right flank s.c.

For tumor rechallenge, the surviving mice after treatment were injected with approximately 1×10^5^ B16F10 melanoma cells intravenously on day 60 after cryo-thermal therapy. Lungs were collected three weeks after the rechallenge.

The animal study protocol was approved by the Ethics Committee of School of Biomedical Engineering and Med-X Research Institute, Shanghai Jiao Tong University (No.2020017).

### The cryo-thermal therapy procedures

The innovative system that achieves cryo-thermal therapy was developed previously for treating local tumors, including successively liquid nitrogen (LN_2_) cooling and radiofrequency ablation. Randomly divided mice with a similar volume of tumor (diameter nearly 10 mm, about 12 days post modeling) were anesthetized with 5% chloral hydrate (Sinopharm, Shanghai, China), and treated with predeterminate parameters according to our experience to entirely ablate the primary tumor (5). Briefly, tumors were frozen at -20°C for 5 minutes by using liquid nitrogen, followed by radiofrequency heating with the temperature 50°C for 10 mins. The primary tumor was entirely ablated after cryo-thermal therapy.

### IFNγ neutralization

For *in vivo* IFNγ neutralization, mice were intraperitoneally (i.p.) injected with anti-IFNγ 250 µg (in 100 µL PBS) monoclonal antibody (Clone XMG1.2, Biolegend, San Diego, CA, USA) on day 5 post treatment.

### Cells isolation

The spleens were collected from different groups at indicated times after cryo-thermal therapy. Single-splenocyte suspension was acquired for immune-cell isolation. EasySep Mouse CD4 Positive Selection Kit II (Cat# 18952, StemCell Technologies, Vancouver, BC, Canada) were used for CD4^+^ T cell isolation. For MDSCs isolation, GR-1 positive cells were labeled with APC by monoclone antibody (Biolegend, San Diego CA), and subsequently selected with the EasySep Mouse APC Positive Selection Kit II (Cat# 17667, StemCell Technologies) following the given instructions from the manufacturer.

### *In vitro* immune cells culture assay

To test whether IFNγ-educated MDSCs regulate the differentiation of CD4^+^ T cells, splenic MDSCs were obtained two weeks post cryo-thermal therapy or cryo-thermal therapy with IFNγ neutralization, and CD4^+^ T cells were isolated from the spleen of tumor-bearing control mice respectively. The CD4^+^ T cells were activated by anti-CD3 (1 µg/mL). 10% of mouse serum from corresponding groups was supplemented to simulate the *in vivo* environment.

To verify whether IFNγ-educated MDSCs regulate the differentiation of CD4^+^ T cells via IL-1β, splenic MDSCs and CD4^+^ T cells were isolated two weeks post cryo-thermal therapy. Subsequently, cells were cocultured in a 1:1 ratio with either the isotype or the anti-IL-1β monoclonal antibody (clone B122, 5 µg/mL, Bio X Cell, West Lebanon, NH, USA). The CD4^+^ T cells were activated by anti-CD3 (1 µg/mL). 10% of mouse serum from corresponding groups was supplemented to simulate the *in vivo* environment.

### Flow cytometry

The weight of the spleen was recorded before treatment. Then splenic single-cell suspension was obtained from the indicated groups and precision count beads were added for absolute number counting. Zombie Dye was performed to exclude dead cells before surface marker labeling. True-Nuclear Transcription Factor Buffer Set was used for transcription factor staining. For intracellular cytokine staining, cells were cultured in the presence of cell activation cocktail (containing PMA (phorbol12-myristate-13-acetate), ionomvcin, and protein transport inhibitor (Brefeldin A)) for 4 hours at the recommended concentration by the manufacturer. Then cells were fixed and permeabilized with fixation buffer and intracellular staining permeabilization wash buffer according to the manufacturer’s instructions. All reagents used were listed in **Supplementary Table S1** and all the fluorochrome-conjugated monoclonal antibodies used were listed in **Supplementary Table S2**. Data were collected using BD FACS Aria II cytometer and processed by FlowJo V10. The absolute number of cells was normalized to beads.

### mRNA isolation and real-time PCR

TRIzol and the PrimeScript RT reagent kit (TaKaRa, Otsu, Shiga, Japan) were utilized for RNA extraction and reverse transcription. For measuring the expression of genes, ABI 7900HT sequence detection system (Applied Biosystems, Foster City, CA, USA), along with SDS 2.4 software, was used for data collection purposes. Results were normalized as GAPDH through ΔΔCt method. The list of all primers used can be found in **Supplementary Table S3**.

### Serum collection and ELISA

The serum of mice was collected five days post cryo-thermal therapy or time-paired untreated control. The concentration of IFNγ was quantified using an ELISA Kit (Boster Biological Technology).

### RNA sequencing

Total RNA was obtained from isolated MDSCs and CD4^+^ T cells as former description. The purity and quantity of RNA were assessed using the NanoDrop 2000 spectrophotometer (Thermo Scientific, USA), and the integrity was evaluated using Agilent 2100 Bioanalyzer (Agilent Technologies, Santa Clara, CA, USA). Subsequently, RNA-seq libraries were generated utilizing the VAHTS Universal V6 RNA-seq Library Prep Kit. OE Biotech Co., Ltd. (Shanghai, China) was entrusted with conducting subsequent transcriptome sequencing and analysis.

The GSEA software was utilized to perform enrichment analysis. This analysis involved the utilization of a predefined gene set, where genes were ranked based on their differential expression between the two sample types. The objective was to assess whether there was significant enrichment of the predefined gene set at either the upper or lower end of the ranking list.

### Statistics analysis

All data were presented as mean ± SD. Statistical analyses were performed using GraphPad Prism V.9.0 (La Jolla, California, USA, https://www.graphpad.com accessed on June 28, 2023). Statistical distinctions in mean values between the two groups were performed using the student’s t-test. For more groups, the differences were measured by one-way analysis of variance (ANOVA) with the Holm-Sidak method. The survival curves were compared using the Kaplan–Meier method and the log-rank test. *P < 0.05, **P < 0.01, ***P < 0.001 and ****p < 0.0001 were considered statistically significant.

## Results

### Cryo-thermal therapy mainly activated IFNγ-producing CD4^+^ Th1 response at the early stage after treatment

**Figure 1:**
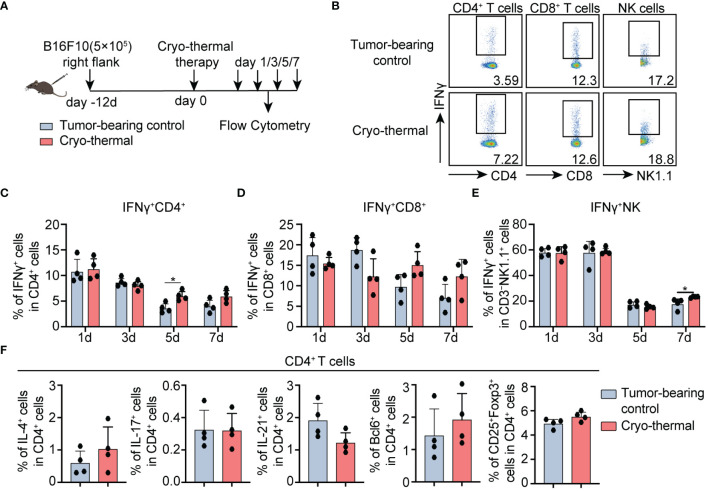
Phenotype of CD4^+^ T cells, CD8^+^ T cells, and NK cells after cryo-thermal therapy. **(A)** Scheme of study design. Briefly, CD4^+^ T cells, CD8^+^ T cells, and NK cells from tumor-bearing mice and cryo-thermal treated mice were detected by flow cytometry at indicated times. **(B–E)** The representative figure of gating strategy **(B)**, the expression of IFNγ in **(C)** CD4^+^ T cells, **(D)** CD8^+^ T cells, **(E)** and NK cells from tumor-bearing control (gray) and cryo-thermal therapy (red). **(F)** The subsets of CD4^+^ T cells on day 5 after cryo-thermal therapy. Experiments were independently repeated at least three times. *p < 0.05. n=4 for each group.

CD4^+^ Th1-dominant immune response rapidly elicited by cryo-thermal therapy was observed in tumor patients with metastasis post treatment in former researches (16). However, whether the upregulated level of early-phase IFNγ, the CD4^+^ Th1-related cytokine, regulates the subsequent differentiation of CD4^+^ T cells remains unknown. Therefore, the proportion of immune cells including CD4^+^, CD8^+^ T cells, and NK cells, the major IFNγ producers, were analyzed in the B16F10 tumor model at the early stage (1, 3, 5, 7d) post ablation (**Figure 1A**). Five days after cryo-thermal therapy, results presented higher frequency and expressive capacity (MFI) of IFNγ^+^ in CD4^+^ T cells after treatment (**Figures 1B, C**; **Supplementary Figures S2C, D**). However, the level of TNFα, which is also one of the markers of T cell activation (17), was similar to the untreated group (**Supplementary Figure S1**). Consistently, both the percentage and absolute numbers of IFNγ^+^ CD4^+^ T cells were also significantly increased (**Supplementary Figures S2A, B**). However, the percentage and expression level of IFNγ in CD8^+^ T cells at the same period were not clearly changed (**Figures 1B, D**; **Supplementary Figures S2C, D**), despite the increased percentage and absolute number of IFNγ^+^CD8^+^ T cells (**Supplementary Figures S2A, B**). The percentage and absolute number of IFNγ in NK cells were unchanged (**Supplementary Figures S2A, B**), with only a slightly upregulated percentage on day 7 (**Figures 1B, E**).

Meanwhile, the frequency of Th2 (IL-4^+^), Th17 (IL-17^+^), Tfh (characterized as the typical transcriptional factor Bcl6, and effective cytokine IL-21) prone cells, and Tregs (CD25^+^Foxp3^+^), were not obviously changed on day 5 (**Figure 1F**; **Supplementary Figures S2E, F**). These collectively indicated that IFNγ-producing CD4^+^ T cells were primarily induced at the early stage after cryo-thermal therapy (on day 5).

### IFNγ induced at early-phase mediated powerful antitumor immunity after cryo-thermal therapy

**Figure 2:**
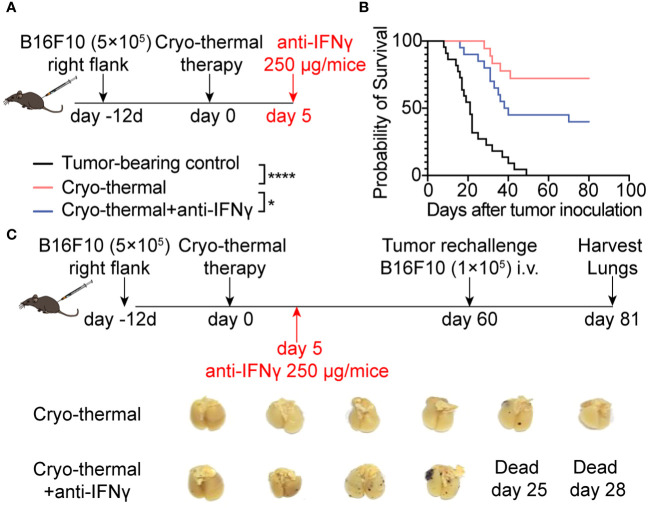
IFNγ at the early stage after cryo-thermal therapy mediated antitumor immune memory and led to a better prognosis. **(A)** Scheme of the experiment design. **(B)** Kaplan–Meier survival curve of tumor-bearing control, cryo-thermal therapy, or cryo-thermal therapy with IFNγ neutralization. The survival curves were compared using log-rank tests. *p < 0.05. ****p < 0.0001. n=22 for each group. **(C)** Scheme of tumor rechallenge design. The treated mice were injected with 1×10^5^ B16F10 cells intravenously on day 60 after cryo-thermal therapy. The lungs were collected on day 21 after the rechallenge. n=6 for the cryo-thermal group. n=4 for the cryo-thermal + anti-IFNγ group for two mice that died before tumor rechallenge.

IFNγ is one of the major effector cytokines of CD4^+^ Th1 cells (1). The above results indicated that a CD4^+^ Th1-prone response was quickly elicited by cryo-thermal therapy (on day 5). At the same time, a higher level of serum IFNγ was observed than the tumor-bearing control group (**Supplementary Figure S2G**). To verify whether IFNγ at the early stage played a major role in cryo-thermal therapy-induced persistent antitumor immune memory, 250 µg anti-IFNγ mAb was administrated on day 5 to neutralize IFNγ (**Figure 2A**). **Figure 2B** presented the significantly decreased survival rate of mice when IFNγ was depleted. Furthermore, these survived mice showed more clear lung metastatic nodules than cryo-thermally treated mice after rechallenge (**Figure 2C**). Taken together, these highlighted the IFNγ presence during cryo-thermal therapy-activated early-phase immune response established subsequent robust antitumor immunity, which effectively suppresses metastasis, thereby resulting in long-term survival of mice.

### Early-phase abundant IFNγ regulated CD4^+^ T cells polarization and other immune cell function

**Figure 3:**
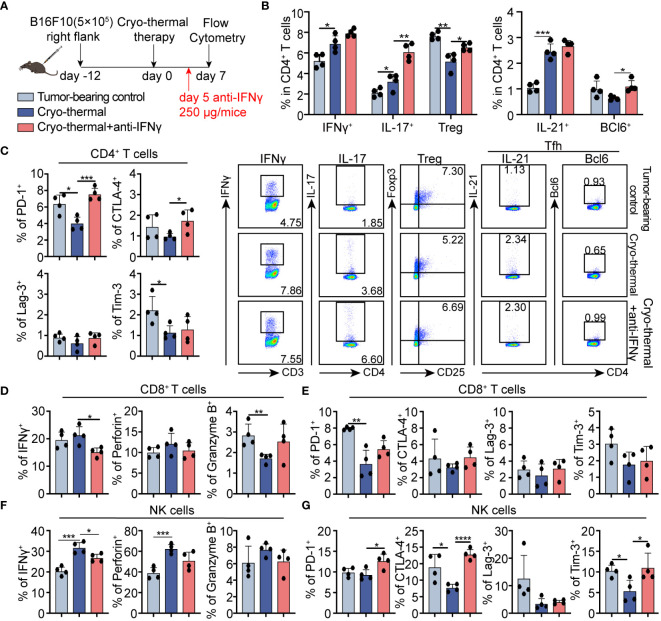
Phenotype of CD4^+^ T cells, CD8^+^ T cells, and NK cells after IFNγ *in vivo* neutralization at the early stage. **(A)** Scheme of study design. 250 µg anti-IFNγ antibodies was injected i.p. on day 5 after cryo-thermal therapy, the phenotype of immune cells was detected by flow cytometry on day 7 post cryo-thermal therapy. **(B, C)** The subsets **(B)** and the immune checkpoints **(C)** of CD4^+^ T cells. **(D, E)** The cytotoxic molecules **(D)** and the immune checkpoints **(E)** of CD8^+^ T cells. **(F, G)** The cytotoxic molecules **(F)** and the immune checkpoints **(G)** of NK cells. Experiments were independently repeated at least three times. *p < 0.05, **p < 0.01, ***p < 0.001, ****p < 0.0001. n=4 for each group.

To validate whether the abundant serum IFNγ mediated the CD4^+^ Th1-dominant response at the early phase after treatment, IFNγ neutralization was performed *in vivo* and the phenotype of immune cells was analyzed two days later (**Figure 3A**). Compared to tumor-bearing controls, the percentage of IFNγ^+^ CD4^+^ Th1-prone cells after cryo-thermal therapy was higher than that of other CD4^+^ T subsets, although the increased percentages of Th1, Th17, Tfh prone CD4^+^ T cells were observed on day 7 after cryo-thermal therapy. Meanwhile, the percentage of Tregs was clearly reduced (**Figure 3B**). After IFNγ neutralization, Th17, Tfh, and Tregs prone CD4^+^ T cells were further increased, but no clear difference in Th1-prone CD4^+^ T cells was observed in comparison with the group that received cryo-thermal therapy (**Figure 3B**). Meanwhile, there were no significant changes observed in the proportion of Th2-prone CD4^+^ T cells and the producing capacity of cytotoxic molecules in CD4^+^ T cells (**Supplementary Figures S3A, B**). These revealed that early-phase high levels of IFNγ after cryo-thermal therapy could inhibit subsequent differentiation patterns, thereby maintaining long-lasting CD4^+^ Th1-dominant immunity.

The expression of inhibitory receptors on CD4^+^ cells was also assessed at the same time point. In comparison to the untreated control, PD-1 and Tim-3 expressed CD4^+^ T cells were downregulated after treatment. In contrast, the levels of PD-1 and CTLA-4 positive cells were significantly increased in the neutralization group compared to cryo-thermal therapy alone. (**Figure 3C**; **Supplementary Figure S4A**). These emphasized the crucial role of IFNγ in mediating CD4^+^ Th1-dominant response and functional activation by inhibiting the differentiation of immunosuppressivors, especially Tregs, and by relieving the co-inhibitory signals.

Moreover, in comparison to the tumor-bearing controls, the PD-1 expressive CD8^+^ T cells was notably decreased, accompanied by a slightly reduced level of granzyme B after cryo-thermal therapy, indicating that CD8^+^ T cell-activation function of cryo-thermal therapy (**Figures 3D, E**; **Supplementary Figure S4B**). After IFNγ neutralization, the expression level of IFNγ in CD8^+^ T cells was notably downregulated (**Figure 3D**; **Supplementary Figure S4B**). However, no clear changes in immune checkpoint inhibitors on CD8^+^ T cells were observed after IFNγ neutralization. These supported the role of IFNγ in CD8^+^ T cell activation of cryo-thermal therapy, with the mechanism of early-phase serum IFNγ promoted the further expression of IFNγ in CD8^+^ T cells.

Furthermore, in NK cells, the generation capacity of IFNγ and perforin were dramatically increased on day 7 after treatment, with downregulated levels of CTLA-4 and Tim-3 compared to those in the tumor-bearing controls (**Figures 3F, G**; **Supplementary Figure S4C**), suggesting the activation and improved cytotoxicity after cryo-thermal therapy. However, following IFNγ neutralization, the expression level of IFNγ in NK cells exhibited a remarkable decrease, while the expression levels of PD-1, CTLA-4, and Tim-3 in NK cells were significantly increased compared to those observed in the cryo-thermal therapy group (**Figures 3F, G**; **Supplementary Figure S4C**). No clear differences in myeloid cells and B cells proportion and phenotype were observed after IFNγ neutralization (**Supplementary Figures S3C–G**). Generally, these supported that IFNγ at the early stage after cryo-thermal therapy is critical for promoting CD4^+^ Th1-dominant differentiation and NK cell activation through coinhibitory signal relief.

### Early-phase abundant IFNγ after cryo-thermal therapy promoted subsequent CD4^+^ Th1-dominant response and other immune cells maturation at the late stage

**Figure 4:**
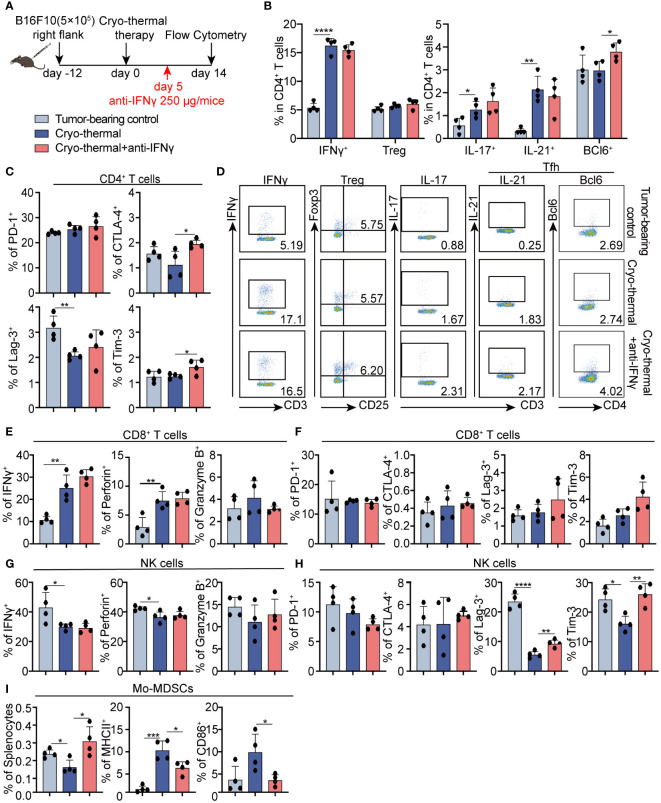
Phenotype of CD4^+^ T cells, CD8^+^ T cells, and NK cells after IFNγ neutralization at the late stage *in vivo*. **(A)** Scheme of study design. Briefly, 250 µg anti-IFNγ antibodies was injected i.p. on day 5 after cryo-thermal therapy, the phenotype of immune cells was detected by flow cytometry on day 14 post cryo-thermal therapy. **(B–D)** The subsets **(B, D)** and the immune checkpoints **(C)** of CD4^+^ T cells. **(E, F)** The cytotoxic molecules **(E)** and the immune checkpoints **(F)** of CD8^+^ T cells. **(G, H)** The cytotoxic molecules **(G)** and the immune checkpoints **(H)** of NK cells. **(I)** The proportion of MDSCs and the expression levels of MHCII and CD86 on MDSCs. Experiments were independently repeated at least three times. *p < 0.05, **p < 0.01, ***p < 0.001, ****p < 0.0001. n=4 for each group.

Results from our earlier research demonstrated that cryo-thermal therapy-induced CD4^+^ Th1-dominant immunity at the memory phase plays a pivotal role in orchestrating long-term antitumor immune responses (6). Whether the early-phase Th1-prone CD4^+^ T cell response demonstrated in the above results helps the maintaining of CD4^+^ Th1-dominant differentiation needs further study. Therefore, the phenotype of CD4^+^ T cells was analyzed at the late stage (two weeks after treatment) by using flow cytometry (**Figure 4A**). The increased percentages of Th1, Th17, and Tfh prone CD4^+^ T cells were observed, but the percentage of Th1-prone CD4^+^ T cells was markedly higher than that of other subsets after cryo-thermal therapy. Meanwhile, no clear changes in Tregs were observed (**Figures 4B, D**). Moreover, there was a decrease in Lag-3 expression on CD4^+^ T cells following cryo-thermal treatment (**Figure 4C**; **Supplementary Figure S5A**). After IFNγ neutralization, a notable increase in Tfh was noted, yet the proportions of Th1 and Th17 prone CD4^+^ T cells, along with Tregs, remained unchanged in comparison to those post-cryo-thermal therapy (**Figures 4B, D**). Furthermore, there was a noted elevation in the expression of CTLA-4 and Tim-3 on CD4^+^ T cells (**Figure 4C**; **Supplementary Figure S5A**). The results suggested that early-phase IFNγ after cryo-thermal therapy maintained subsequent CD4^+^ T cell activation and the Th1-dominant immunity by impairing the differentiation of Tfh and downregulating the expression of checkpoint inhibitors at the late stage.

Furthermore, cytotoxic cytokines and immune checkpoint molecules on CD8^+^ T cells and NK cells were also detected at the late stage. On day 14, there was a significant increase in the expression levels of IFNγ and perforin in CD8^+^ T cells induced by cryo-thermal therapy (**Figure 4E**; **Supplementary Figure S5B**). However, the levels of ICs were not changed (**Figure 4F**; **Supplementary Figure S5B**). The expression levels of cytotoxic cytokines and immune checkpoint inhibitors were all not clearly changed after IFNγ neutralization at the early stage (**Figures 4E, F**; **Supplementary Figure S5B**), suggesting that IFNγ at the early stage after treatment had less of an effect on CD8^+^ T cell immunity at the late stage.

Moreover, the decreased levels of IFNγ and perforin in NK cells, and immune checkpoint inhibitors Lag-3 and Tim-3 on NK cells were observed on day 14 after treatment (**Figures 4G, H**; **Supplementary Figure S5C**), while also no impact on cytotoxic cytokine levels when IFNγ neutralization (**Figures 4G**; **Supplementary Figure S5C**). However, the levels of Lag-3 and Tim-3 in NK cells were upregulated (**Figure 4H**; **Supplementary Figure S5C**). These data revealed that early-phase IFNγ facilitated later activation of NK cells.

Interestingly, the population of monocytic (Mo)-MDSCs was clearly downregulated, along with the increased expression of MHCII and CD86 at the same time as compared to those in tumor-bearing control (**Figure 4I**; **Supplementary Figures S5D, E**), indicating that cryo-thermal therapy promoted Mo-MDSCs maturation. However, after IFNγ neutralization at the early stage, Mo-MDSC was significantly expanded, accompanied by the downregulated level of MHCII and CD86 (**Figure 4I**; **Supplementary Figure S5D**). These results revealed that early phase IFNγ was crucial for thereby Mo-MDSCs maturation after cryo-thermal therapy. Briefly, early-phase IFNγ after cryo-thermal therapy maintained subsequent CD4^+^ Th1-dominant differentiation, facilitated Mo-MDSCs maturation and NK cell activation at the late stage.

### IFNγ at the early stage promoted the generation of IL-1β in MDSCs, which amplify CD4^+^ Th1-dominant differentiation

**Figure 5:**
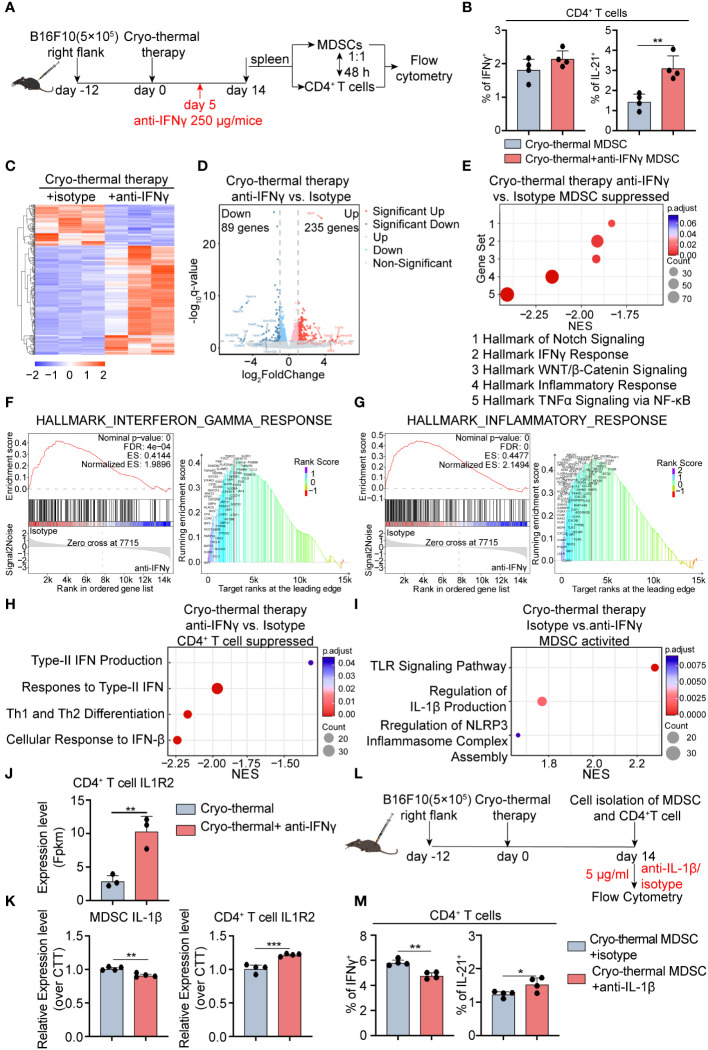
Transcriptomic profile of MDSCs and IL-1β secreted by MDSCs are involved in maintaining CD4^+^ Th1-dominated differentiation. **(A)** Scheme of *in vitro* design. MDSCs from cryo-thermal therapy and cryo-thermal therapy with IFNγ neutralization on day 14 were isolated by MASC and cocultured with CD4^+^ T cells from tumor-bearing control (0d). After 48 hours, the subsets **(B)** of CD4^+^ T cells were analyzed by flow cytometry. MDSCs and CD4^+^ T cells were isolated by MACS and analyzed by RNA-seq. **(C)** Heatmap of differentially expressed genes. **(D)** Volcano plot of gene expression change of MDSCs from cryo-thermal therapy injected with anti-IFNγ antibody over cryo-thermal treated therapy. **(E)** GSEA analysis showing significantly suppressed gene sets of MDSCs cryo-thermal with IFNγ neutralization mice. **(F)** Individual GSEA enrichment plot for the hallmark inflammation response gene set (left), GSEA dot lot (right). **(G)** Individual GSEA enrichment plot for the hallmark IFNγ response gene set (left), GSEA dot plot (right). n=3 for each group in RNA-seq. **(H)** GSEA analysis showing significantly suppressed gene sets of CD4^+^ T cells cryo-thermal with IFNγ neutralization mice. **(I)** GSEA analysis showing significantly activated gene sets of MDSCs from cryo-thermal therapy. **(J)** The expression level of IL-1R2 on CD4^+^ T cells from RNA-seq. **(K)** The expression levels of IL-1β in MDSCs and IL-1R2 on CD4^+^ T cells from cryo-thermal therapy and cryo-thermal therapy with IFNγ neutralization were detected by RT-PCR. **(L)** Scheme of *in vitro* design. MDSCs from the cryo-thermal therapy on day 14 were isolated by MASC and cocultured with CD4^+^ T cells from cryo-thermal therapy (14d) in the presence of 5 µg/mL isotype or the anti-IL-1β antibody. Forty-eight hours, the subsets **(M)** of CD4^+^ T cells were analyzed by flow cytometry. In both *in vitro* experiments, the corresponding serum was administered to both groups to mimic the *in vivo* environment, and an anti-CD3 agonist (1 µg/mL) was added to stimulate the activation of CD4^+^ T cells. Experiments were independently repeated at least three times. *p < 0.05, **p < 0.01, ***p < 0.001. n=4 for each group *in vitro* experiments.

The above results showed that IFNγ at the early stage promoted subsequent MDSC maturation. MDSCs modulate the differentiation of CD4^+^ T cells into diverse functional subsets (18). To verify the role of MDSCs regulated by IFNγ present during the early stage in thereby CD4^+^ T cell differentiation, MDSCs were isolated from cryo-thermal therapy, and cryo-thermal therapy with IFNγ neutralization and co-cultivated with control mice-derived CD4^+^ T cells (0d) *in vitro* for 48h (**Figure 5A**). In comparison to cryo-thermal therapy, there were no clear changes in the proportion of Th1 prone cells and Tregs after IFNγ neutralization, but MDSCs from cryo-thermal therapy with IFNγ neutralization facilitated Tfh prone polarization (**Figure 5B**; **Supplementary Figures S7A, B**), which was in accordance with *in vivo* studies (**Figure 4B**). These results suggested that IFNγ at the early stage promoted the maturation of MDSCs into potent antigen-presenting cells (APCs), thereby leading to CD4^+^ Th1-dominant differentiation while inhibiting Tfh differentiation.

To understand the in-depth role of early-phase IFNγ in regulating the maturation of MDSCs at the late stage after treatment, we investigated how MDSCs modulated CD4^+^ Th1-dominant differentiation, and RNA-seq was conducted to assess gene expressive levels in both MDSCs and CD4^+^ T cells on day 14, respectively. In comparison to cryo-thermal therapy, MDSCs from the IFNγ neutralization group displayed an altered transcriptomic profile, in which a total of 324 differential genes were found, including 235 upregulated and 89 downregulated genes (**Figures 5C, D**). GSEA showed that the top 5 pathways in MDSCs administered cryo-thermal therapy with IFNγ neutralization were significantly inhibited and were all involved in inflammatory-related (tumor necrosis factor-alpha (TNFα) signaling, Inflammatory response, Wnt/β catenin signaling, Notch signaling) and IFNγ response pathways (**Figures 5E–G**). Phagocytosis is enhanced in myeloid cells primed by TNFα (19). Inflammatory response-related genes, such as Toll-like receptor (TLR), and Notch signaling pathways, are upregulated in MDSCs, which means that MDSCs differentiate into mature APCs (20, 21). The response to IFNγ in MDSCs dampens the immunosuppressive function of MDSCs by preventing the PD-1 signaling pathway (22). The Wnt/β-Catenin signaling pathway tightly regulates the inflammatory response to prevent tissue injury caused by uncontrolled inflammation (23). These suggested that cryo-thermal therapy-induced early-phase IFNγ neutralization restrained the phagocytic capacity of MDSCs as well as their differentiation into APCs. Simultaneously, the development of chronic inflammation was induced by inhibiting Wnt/β-catenin signaling, ultimately enhancing the immunosuppressive function of MDSCs. In addition, GSEA showed that signaling pathways related to CD4^+^ Th1 differentiation (Th1, cellular response to Interferon-Beta, Response to type II Interferon, Type II Interferon production) were significantly inhibited in CD4^+^ T cells in the neutralization group (**Figure 5H**). The responsiveness to type I IFN promotes Th1-like differentiation of CD4^+^ T cells (24). Furthermore, autocrine IFNγ promotes T-bet expression which establishes the positive loop to further promote IFNγ generation (25). These results suggested that Th1 prone CD4^+^ T cell differentiation is impeded after early-phase IFNγ neutralization, leading to a diminishing capacity to produce IFNγ.

GSEA showed that the TLR signaling pathway, regulation of IL-1β production, and regulation of NOD-like receptor family pyrin domain-containing 3 (NLRP3) inflammasome complex assembly pathway were markedly upregulated in MDSCs after cryo-thermal therapy (**Figure 5I**). TLR signal primes the expression of pro-IL-1β, NLRP3, and caspase-1 in the cytosol (26). Then, myeloid cells secret IL-1β to promote Th1 prone CD4^+^ T cell differentiation when NLRP3 is activated in myeloid cells (27). Moreover, the expression of TLRs related to the inflammatory response was downregulated after cryo-thermal treatment with IFNγ neutralization (**Figures 5E, F**). At the same time, RNA-Seq showed downregulated level of CD4^+^ T cell-IL-1R2, which can competitively bind IL-1β from binding to IL-1R1 to negatively regulate IL-1β signaling (28), compared to the anti-IFNγ performed group (**Figure 5J**). Then, we hypothesized that the cryo-thermally induced IFNγ-abundant environment early after treatment led to the activation of TLRs on MDSCs. Subsequently, TLR on MDSCs triggered the activation of genes linked to the NLRP3 pathway, leading to the eventual secretion of mature IL-1β. Moreover, the low expression level of IL-1R2 on CD4^+^ T cells guaranteed their responsiveness to IL-1β, thereby leading to Th1 prone differentiation promoted by mature MDSCs. Therefore, we suggested that MDSCs are reprogrammed by early-phase IFNγ, in turn maintaining Th1 response via IL-1β secreted by mature MDSCs at the late stage. Consistent with RNA-seq results, a decreased expression level of IL-1β in MDSCs and an increased level of IL-1R2 on CD4^+^ T cells were confirmed by qRT-PCR after IFNγ neutralization (**Figure 5K**).

Then, separated splenic MDSCs were co-cultivated with CD4^+^ T cells in the presence of isotype control or anti-IL-1β antibody (**Figure 5L**). A decreased percentage of Th1 prone cells with an increased ratio of Tfh was observed when IL-1β signaling blockade *in vitro* (**Figure 5M**; **Supplementary Figure S7C**), aligning with findings from *in vivo* study (**Figure 4B**). The finding indicated that early-phase IFNγ promoted MDSCs maturation and induced MDSC-secreted IL-1β, thereby preserving the predominance of CD4^+^ Th1-dominant differentiation post cryo-thermal treatment.

## Discussion

Previously, we revealed that cryo-thermal therapy elicited CD4^+^ Th1-dominant differentiation at the late stage in the B16F10 tumor model, thereby leading to long-term survival (6). However, the underlying mechanism responsible for the maintenance of CD4^+^ Th1-dominant immunity remains elusive. This research demonstrated that cryo-thermal therapy elicited an IFNγ-abundant environment early after treatment, thereby promoting CD4^+^ Th1-dominant immunity and subsequently facilitating MDSC maturation. Then, mature MDSCs maintained CD4^+^Th1 prone differentiation via a high level of IL-1β at the late stage. The present study emphasized the important role of cryo-thermal therapy-induced early-phase IFNγ in maintaining CD4^+^ Th1-dominated differentiation through mature MDSCs secreted IL-1β at the late stage, which could be an early biomarker for better prognosis.

CD4^+^ T cells exhibit high plasticity in polarizing into different subsets (29), while the balance between anti- or pro-tumorigenic CD4^+^ T cells determines the outcome (1). CD4^+^ Th1 exert a strong antitumor immune response (30). IFNγ, the hallmark cytokine of the CD4^+^ Th1 subset, induces T-bet generation to stabilize CD4^+^ Th1 differentiation (31) and promotes the antitumor function of other subsets. However, Tregs are important suppressors in the tumor environment (32), while IFNγ prevents the neogeneration of Tregs through reactive oxygen species (ROS)-mediated apoptosis (33), and counteracts IL-10 and Transforming growth factor-β (TGF-β) signaling-mediated inhibition (34). Abundant TGF-β production derived by Tregs directly inhibits the function of IFNγ, thereby preventing CD4^+^ Th1 differentiation (35, 36). These results suggested that inducing CD4^+^ Th1-dominant antitumor immunity and inhibiting Tregs differentiation are key for better efficacy of tumor treatment. Here, cryo-thermal therapy triggered CD4^+^ Th1 differentiation and decreased Tregs proportion, while IFNγ neutralization significantly facilitated the expansion of Tregs, indicating that early-phase IFNγ after cryo-thermal therapy could regulate the balance between CD4^+^ Th1 cells and Tregs early after treatment, thereby influencing the subsequent immune response.

MDSCs are a diverse group of immature myeloid cells known for their strong immunosuppressive effects. However, MDSCs possess the capacity to undergo conversion into immunostimulatory cells (37). IFNγ reduces the inhibitory function of MDSCs through the decreased expression of arginase1 (Arg1) and Programmed death ligand 2 (PD-L2) (22, 38). In our previous study, IFNγ triggers MDSCs maturation marked by upregulated expression of MHCII (39). This study revealed an increase in MHCII and CD86 expression on MDSCs following cryo-thermal treatment, whereas early-stage cryo-thermal therapy using IFNγ neutralization reversed the upregulation. Furthermore, IFNγ-educated MDSCs after cryo-thermal therapy showed enhanced capacity for IL-1β production. IL-1β acts as a multifaceted agent in inflammation, significantly contributing to the activation of the innate immune system and the development of myeloid cells (40). Stimulation via TLR induces the activation of nuclear factor-kappa B (NF-κB) and subsequent NLRP3 response leads to the expression of IL-1β in myeloid cells (26). In this process, IFNγ synergistically activates MyD88, the adaptor of TLR, to enhance the expression of genes downstream of TLR and facilitate the function of myeloid cells (41, 42). Therefore, the expression of IL-1β along with the upregulated antigen-presenting capacity are the characteristics of hyperactivated myeloid cells (43). In this study, enrichment of TLR, NLRP3 complex assembly signaling pathway, and IL-1β production in MDSCs with an improved expression of MHCII and CD86 was induced by cryo-thermal therapy. On the contrary, IFNγ neutralization prevented the production of IL-1β in MDSCs and upregulated IL-1R2 expression on CD4^+^ T cells to additionally inhibit IL-1β signaling. These results illustrate that CD4^+^ Th1-dominant differentiation along with suppressing Tregs differentiation early after cryo-thermal therapy induced the IFNγ-abundant environment, which contributed to the hyperactivation of MDSCs to remodel the suppressive environment and maintained CD4^+^ Th1-dominant antitumor immunity at the late stage.

IL-1β plays a beneficial role in initiating adaptive antitumor responses (44). IL-1β generated by activated APCs induces the frequencies of IFNγ-producing cytotoxic T lymphocytes and CD4^+^ Th1 cells (27, 45). In this study, MDSCs educated by IFNγ enhanced their ability capacity to maintain CD4^+^ Th1 differentiation and reduce the proportion of Tfh in CD4^+^ T cells after cryo-thermal therapy. Our studies highlighted the mechanism of IFNγ in maintaining long-term CD4^+^ Th1-dominant immunity by remodeling the function of MDSCs with the increased IL-1β production after cryo-thermal therapy.

In clinical melanoma, CD4^+^ Th1/IFNγ was found as the biomarker of better prognosis (46, 47). Previously, we also demonstrated the CD4^+^ Th1-dominant immunity at the late stage after cryo-thermal therapy coordinates both innate and adaptive immunity to induce long-lasting tumor cytotoxicity and improve the tumor-free survival of mice (6). Here, we clarified that early-phase IFNγ induced by cryo-thermal therapy was essential for sustaining durable CD4^+^ Th1 immunity. Our studies suggested that early-phase IFNγ could serve as an early biomarker for better efficacy after cryo-thermal therapy.

CD4^+^ Th1 prevents the generation of Th17 cells and Tfh cells (10, 11), aligning with our findings showing a rise in Th17 and Tfh cells when IFNγ neutralization. However, we observed an upregulated ratio of Th17 and Tfh cells accompanied by CD4^+^ Th1-dominant differentiation early after cryo-thermal therapy in this study. Th1 has a strong capacity to trigger antitumor response (3). However, the role of Th17 and Tfh is still unclear. Th17 helps the tumor development through IL-17-induced angiogenesis (1), and inhibits the function of effector T cells (48). But in Th1 abundant environment, IFNγ reprofiles the cytokine expression of Th17, leading to strong tumor elimination capacity in B16 melanoma (49, 50). In addition, there are some studies about the effects of Tfh in tumor control (51, 52) and tumor promoting action (53, 54). In our previous study, T cells are highly activated within 7 days, and stable Th1-dominant differentiation is established on day 14 after treatment in the B16F10 model. T cell differentiation pattern largely depends on the cytokine milieu. Th1 initiation relies on type I IFN and IL-12 signaling to promote the expression of T-bet and IFNγ through STAT1/4, while STAT4 promotes Bcl6 and IL-21 (the markers of Tfh) in early Th1 cells (14). In addition, IL-1β also supports the differentiation of both Th1 and Th17 (55). A high level of IL-1β expression in neutrophils was maintained early after cryo-thermal therapy (unpublished data). Therefore, in this study, the upregulated Th17 and Tfh on day 7 after cryo-thermal therapy would be attributed to the early stage of CD4^+^ Th1 differentiation. In consistent, at the late stage after cryo-thermal therapy (14 days), the proportion of Th1 continued to increase as compared to day 7, while the proportion of Th17 and Tfh was decreased, finally CD4^+^ Th1-dominant differentiation was induced.

In conclusion, we revealed that CD4^+^ Th1-dominant differentiation was elicited by cryo-thermal therapy and established an IFNγ-abundant immunostimulatory environment at the early stage, which promoted the maturation of MDSCs with the increased expression of IL-1β to maintain long-term CD4^+^ Th1 immunity, thereby leading to powerful systematic antitumor immunity. This study highlighted the significance of early-phase IFNγ after cryo-thermal therapy, which could be a biomarker for better tumor prognosis.

## Data availability statement

The original contributions presented in the study are publicly available. This data can be found here: NCBI BioProject is PRJNA105065.

## Ethics statement

The animal study was approved by Shanghai Jiao Tong University Animal Care. The study was conducted in accordance with the local legislation and institutional requirements.

## Author contributions

JW: Writing – original draft, Writing – review & editing, Conceptualization, Data curation, Investigation, Methodology. YL: Conceptualization, Formal analysis, Investigation, Writing – original draft. SW: Methodology, Writing – original draft. ZZ: Methodology, Writing – original draft. JY: Methodology, Writing – original draft. YZ: Methodology, Writing – original draft. YY: Methodology, Writing – original draft. YH: Methodology, Writing – original draft. PL: Writing – review & editing, Conceptualization, Formal analysis, Funding acquisition, Project administration, Supervision. LX: Writing – review & editing, Funding acquisition.
