# Supplementary material for: IFNγ at the early stage induced after cryo-thermal therapy maintains CD4+ Th1-prone differentiation, leading to long-term antitumor immunity
Source: Front Immunol. 2024 May 17;15:1345046. doi: 10.3389/fimmu.2024.1345046 (PMC11140566; doi:10.3389/fimmu.2024.1345046)
Supplement: Supplementary file 1 [file DataSheet_1.docx]

Supplementary Material

**IFNγ induced at the early stage after cryo-thermal therapy maintains CD4^+^ Th1-dominant differentiation, leading to long-term antitumor immunity**

Junjun Wang ^1^, Yue Lou ^1^, Shicheng Wang ^1^, Zelu Zhang ^1^, Jiaqi You ^1^, Yongxin Zhu ^1^, Yichen Yao ^1^, Yuankai Hao ^1^, Ping Liu ^1,^ * and Lisa X Xu ^1,^ *

^1^ School of Biomedical Engineering and Med-X Research Institute, Shanghai Jiao Tong University, Shanghai, China

^*^ Corresponding: Lisa X. Xu: lisaxu@sjtu.edu.cn; Ping Liu: pingliu@sjtu.edu.cn

**Table S1.** Reagents used for flow cytometry detection

| **Reagent** | **Identifier** | **Company** |
| --- | --- | --- |
| Precision count beads | Cat# 424902 | Biolegend^1^ |
| Zombie Dye | Cat# 423102 | Biolegend |
| Cell Activation Cocktail (with Brefeldin A) | Cat# 423304 | Biolegend |
| Fixation buffer | Cat# 420801 | Biolegend |
| Intracellular Staining permeabilization buffer | Cat# 421002 | Biolegend |

^1^ Biolegend, San Diego, CA, USA

**Table S2**. Antibodies used for flow cytometry

| **Antibodies** | **Fluorescence** **Labeling** | **Clone** | **Company** |
| --- | --- | --- | --- |
| CD11b | Pacific Blue | M1/70 | Biolegend^1^ |
| Gr-1 | APC | RB6-8C5 | Biolegend |
| Ly6G | PE/Cy7 | 1A8 | Biolegend |
| Ly6C | FITC | HK1.4 | Biolegend |
| CD19 | APC | 1D3/CD19 | Biolegend |
| F4/80 | APC or BV711 | BM8 | Biolegend |
| CD11c | PE | N418 | Biolegend |
| CD86 | APC/Cy7 or PE/Dazzle 594 | GL-1 | Biolegend |
| MHCII | Percp/Cy5.5 | M5/114.15.2 | Biolegend |
| CD3 | FITC or Percp/Cy5.5 or APC/Cy7 | 145-2C11 | Biolegend |
| CD4 | APC/Cy7 or PE/Cy7 or APC | RM4-5 | Biolegend |
| CD8 | Pacific Blue or AF700 | 53-6.7 | Biolegend |
| NK1.1 | FITC or Percp/Cy5.5 | PK136 | Biolegend |
| PD-1 | PE/Cy7 or PE | 29F.1A12 | Biolegend |
| CTLA-4 | BV421 | UC10-4B9 | Biolegend |
| Lag-3 | BV711 | C9B7W | Biolegend |
| CD25 | PE/Cy7 | 3C7 | Biolegend |
| Foxp3 | PE | MF-14 | Biolegend |
| Bcl6 | BV421 | K112-91 | BD^2^ |
| IFNγ | PE/Dazzle 594 or BV605 or BV510 | XMG1.2 | Biolegend |
| IL-4 | BV421 | 11B11 | Biolegend |
| IL-17A | BV711 or PE | TC11-18H10.1 | Biolegend |
| Perforin | PE | S16009A | Biolegend |
| Granzyme | AF647 or FITC | GB11 | Biolegend |
| Tim-3 | BUV395 | 5d12 | BD |
| IL-21 | APC | FFA21 | eBioscience^3^ |
| TNF-α | FITC | MP6-XT22 | Biolegend |
| CD45 | BUV395 or PE/Cy7 | [30-F11](https://www.biolegend.com/en-us/search-results?Clone=30-F11) | BD & Biolegend |

^1^ Biolegend, San Diego, CA, USA

^2^ BD Biosciences, Franklin Lakes, NJ, USA

^3^ eBioscience, Waltham, MA USA

**Table S3.** Primer sequences of various genes in this study

| **Name** | **Primer Sequence (5'-3')** |
| --- | --- |
| IL-1β-F | ACAGCAGCACATCAACAAGAG |
| IL-1β-R | ATGGGAACGTCACACACCAG |
| IL-1R2-F | GTTTCTGCTTTCACCACTCCA |
| IL-1R2-R | GAGTCCAATTTACTCCAGGTCAG |


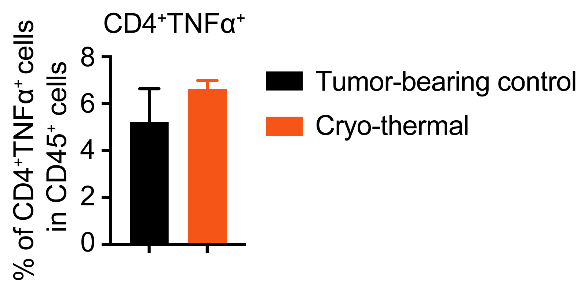


**Figure S1 Expression of TNF-α in CD4^+^ T cells.** Expression of TNF-α in CD4^+^ T cells was detected on day 5 after cryo-thermal therapy by FACS. n = 4 for each group.


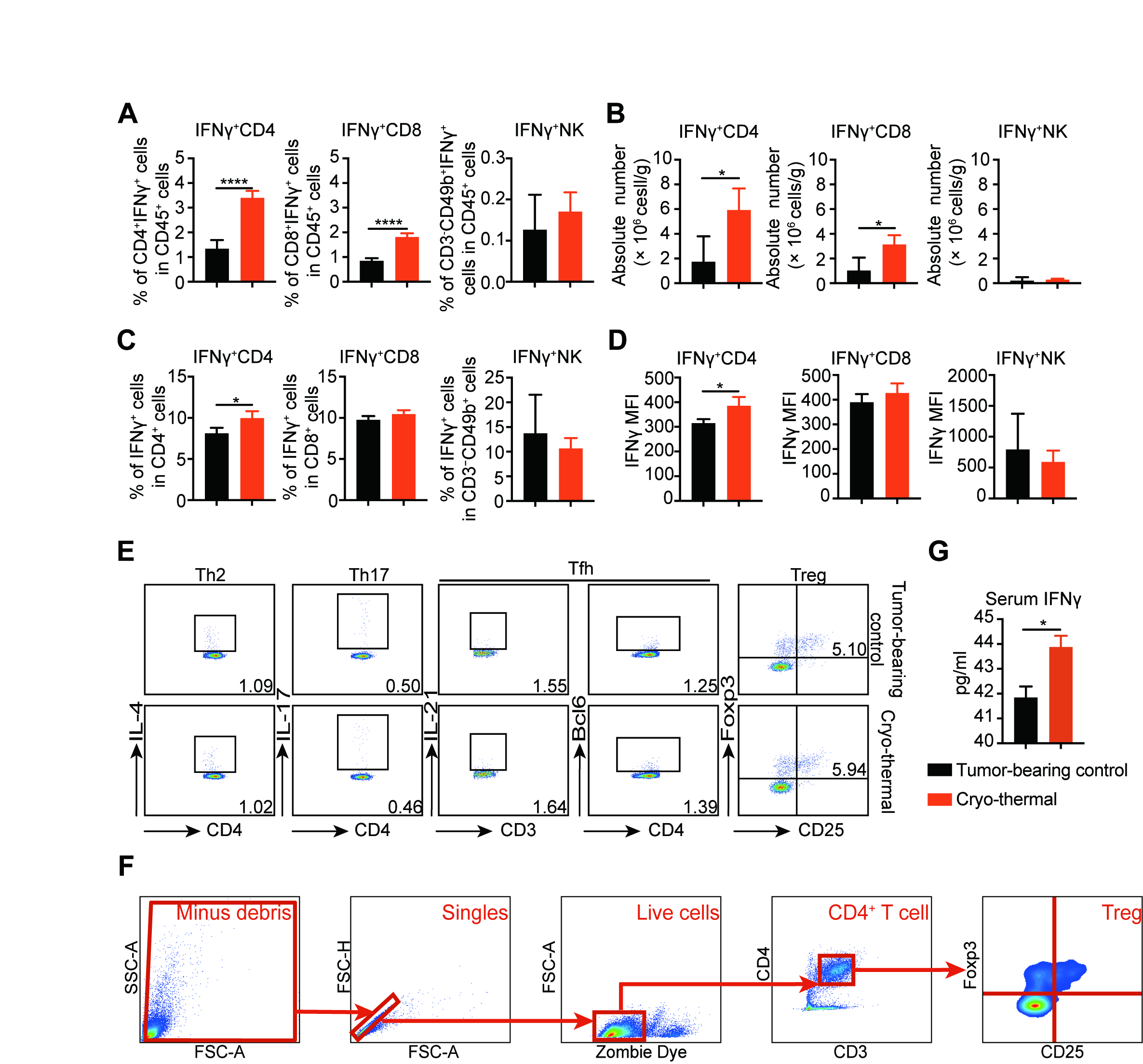


**Figure S2 Absolute number and IFNγ expressing capacity of CD4^+^, CD8^+^ T cells and NK cells.** **(A-D)** The absolute number and percentage of IFNγ expressing cells were detected on day 5 after cryo-thermal therapy. **(A)** Percentage of IFNγ^+^ CD4^+^, CD8^+^ T cells and NK cells in CD45^+^ immune cells. **(B)** Absolute number of IFNγ^+^ CD4^+^, CD8^+^ T cells, and NK cells. **(C)** Percentage of IFNγ expressing CD4^+^, CD8^+^ T cells, and NK cells. **(D)** Mean fluorescence intensity of IFNγ in CD4^+^, CD8^+^ T cells and NK cells. **(E)** The dots plots of Th2 (IL-4^+^), Th17 (IL-17^+^), Tfh (characterized as the typical transcriptional factor Bcl6, and effective cytokine IL-21) cells, and Tregs (CD25^+^Foxp3^+^). **(F)** The gating strategy of CD25^+^Foxp3^+^ cells. **(G)** Serum IFNγ concentration on day 5 after cryo-thermal therapy detected by ELISA. *p < 0.05, ****p < 0.0001. n = 4 for each group.


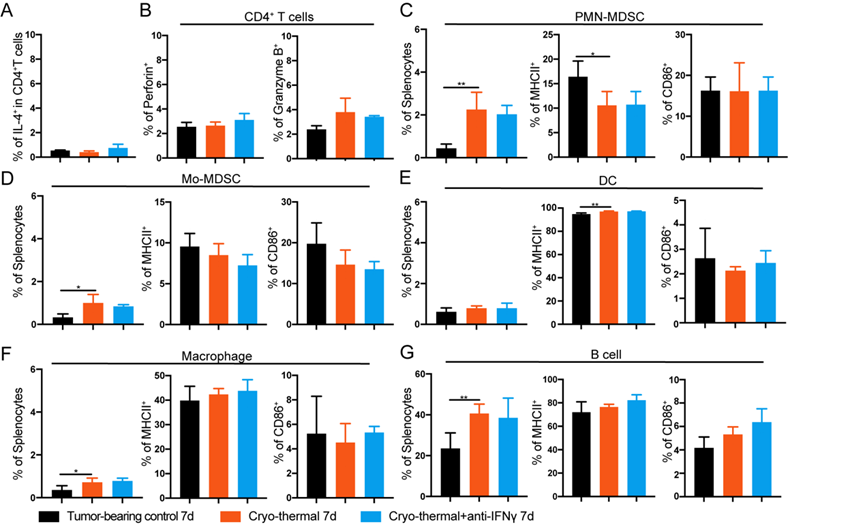


**Figure S3 Phenotype of immune cells at the early stage after cryo-thermal therapy with IFNγ neutralization. (A, B)** The subsets **(A)** and the cytotoxic molecules **(B)** of CD4^+^ T cells. **(C-G)** The proportion and expression levels of MHCII and CD86 on **(C)** PMN-MDSCs, **(D)** Mo-MDSCs, **(E)** Dendritic cells (DCs), **(F)** Macrophages, and **(G)** B cells in the spleen. *p < 0.05, **p < 0.01. n = 4 for each group.


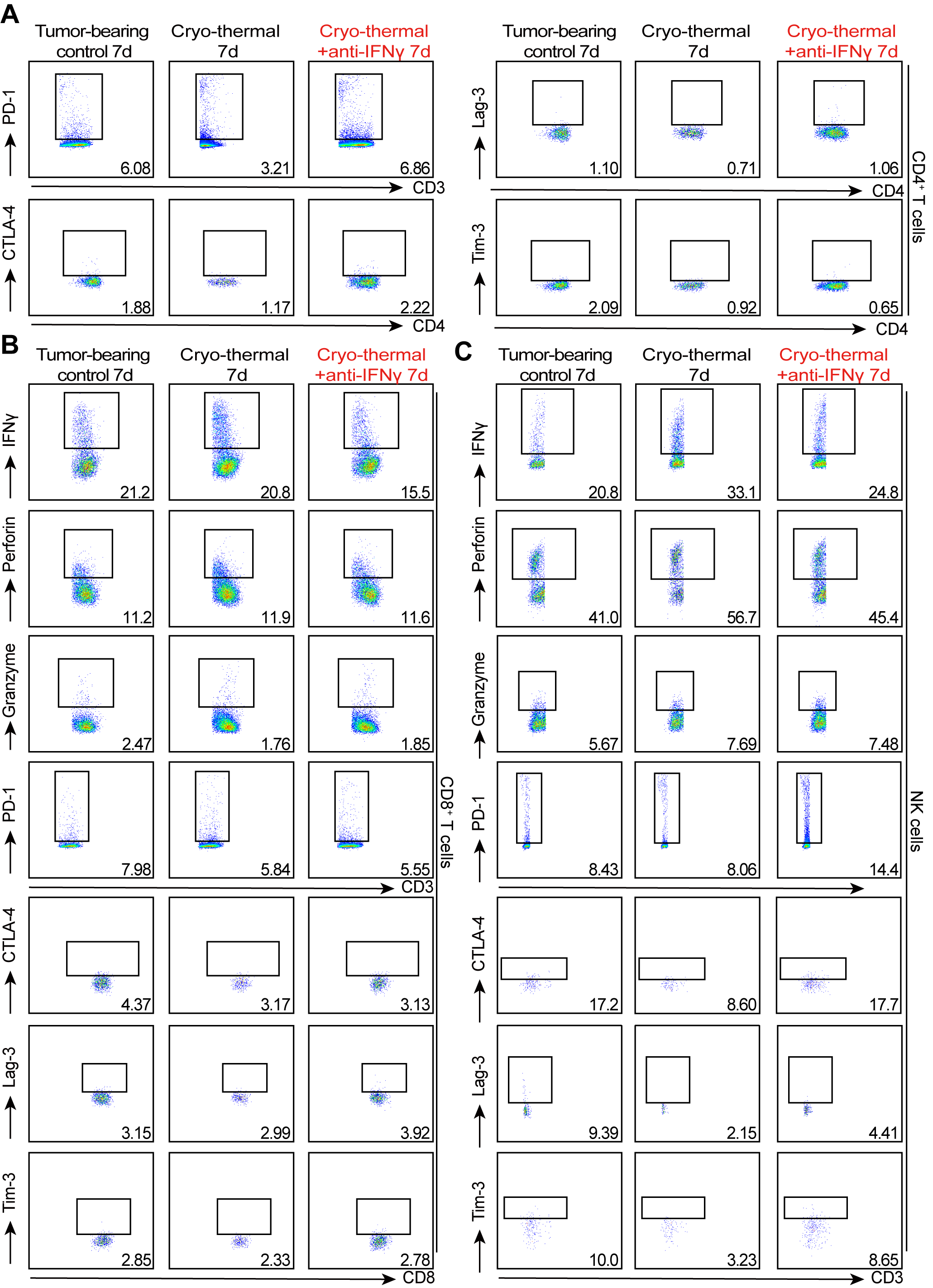


**Figure S4 The dots plots of CD4^+^ T cells, CD8^+^ T cells, and NK cells *in vivo* after IFNγ neutralization on day 7.** **(A)** The representative figures for the immune checkpoint of CD4^+^ T cells. **(B)** The representative figures for the cytotoxic molecules and immune checkpoint of CD8^+^ T cells. **(C)** The representative figures for the cytotoxic molecules and immune checkpoint of NK cells.


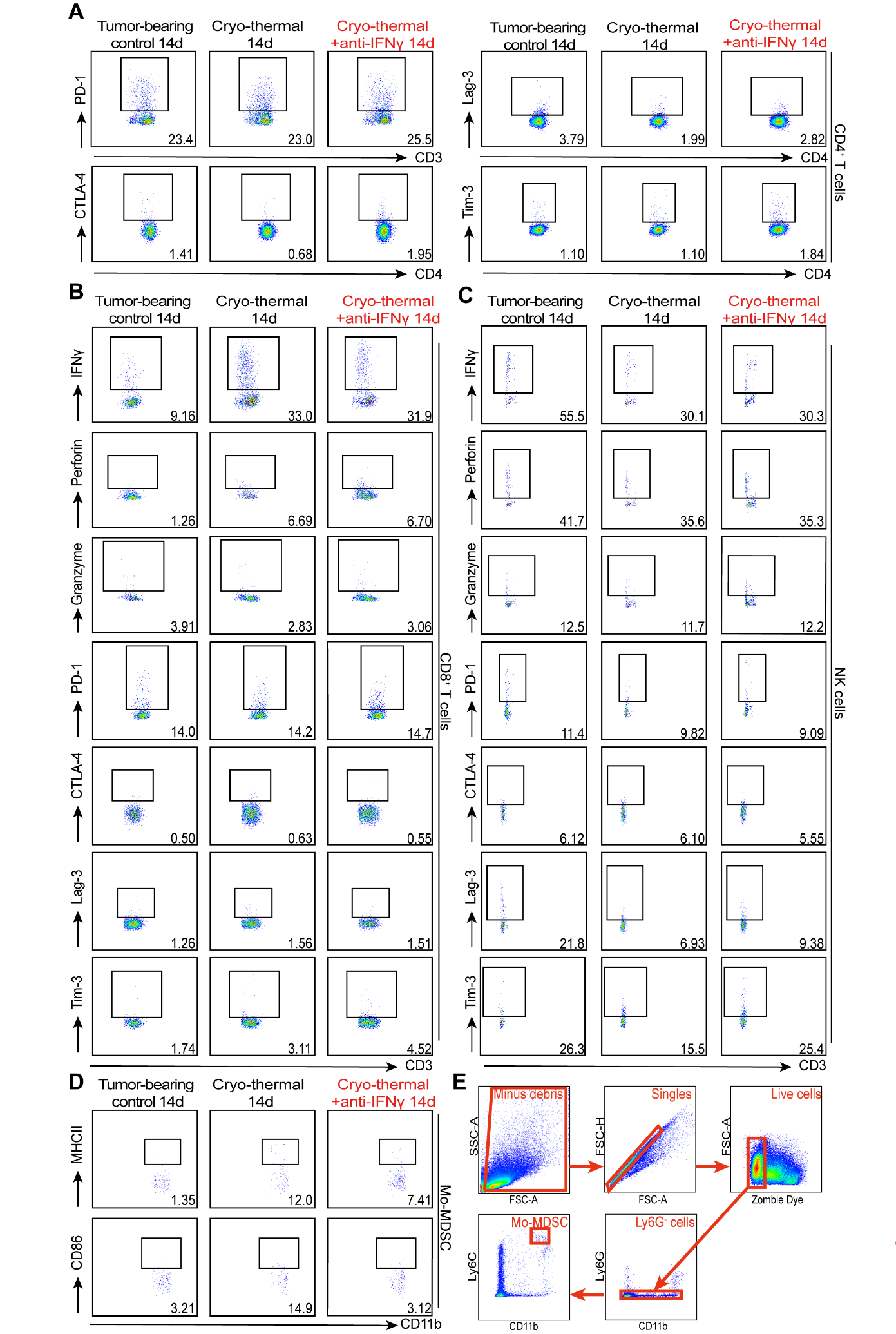


**Figure S5 The dots plots of CD4^+^ T cells, CD8^+^ T cells, NK cells, and Mo-MDSC *in vivo* after IFNγ neutralization on day 14.** **(A)** The representative figures for the immune checkpoint of CD4^+^ T cells. **(B)** The representative figures for the cytotoxic molecules and immune checkpoint of CD8^+^ T cells. **(C)** The representative figures for the cytotoxic molecules and immune checkpoint of NK cells. **(D)** The representative figures for the MHCII and CD86 of Mo-MDSC. **(E)** The gating strategy of Mo-MDSC.
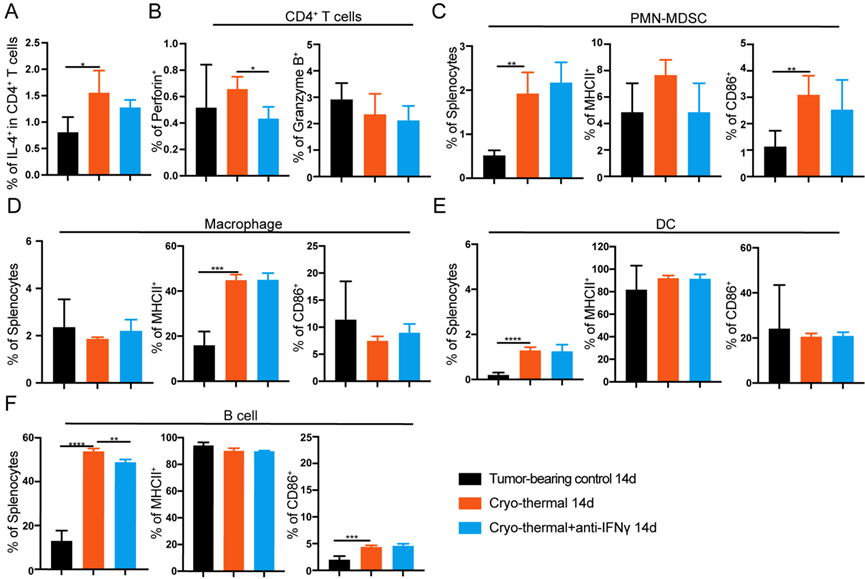


**Figure S6** **Phenotype of immune cells at the late stage after cryo-thermal therapy with IFNγ neutralization.** **(A, B)** The subsets **(A)** and the cytotoxic molecules **(B)** of CD4^+^ T cells. **(C-F)** The proportion and expression levels of MHCII and CD86 in **(C)** PMN-MDSCs, **(D)** Macrophages, **(E)** DCs, and **(F)** B cells in the spleen. *p < 0.05, **p < 0.01, ***p < 0.001, ****p < 0.0001. n= 4 for each group.


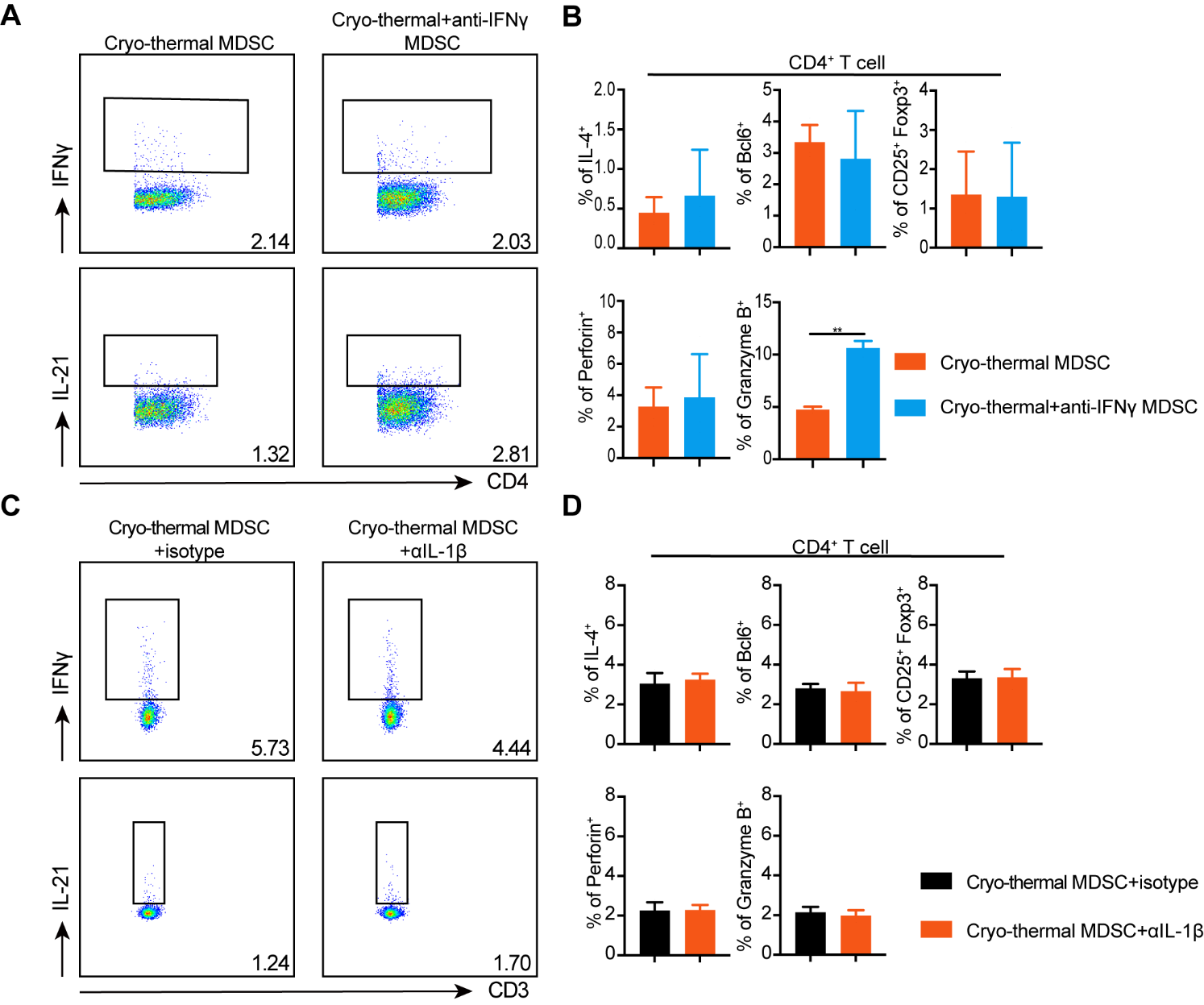


**Figure S7 Phenotype of CD4^+^ T cells cocultured with MDSCs *in vitro*. (A, B)** The subsets of CD4^+^ T cells after coculturing with MDSCs *in vitro*. **(C, D)** The subsets of CD4^+^ T cells after IL-1β neutralization. **p < 0.01. n = 4 for each group.
